# Diagnosis of malaria in pregnancy: accuracy of CareStart™ malaria Pf/PAN against light microscopy among symptomatic pregnant women at the Central Hospital in Yaoundé, Cameroon

**DOI:** 10.1186/s12936-022-04109-6

**Published:** 2022-03-09

**Authors:** Cliford Ebontane Ebong, Innocent Mbulli Ali, Hortence Jeanne Fouedjio, Estelle Essangui, Dorothy Fosah Achu, Ayong Lawrence, Dohbit Sama

**Affiliations:** 1 Department of Gynecology/Obstetrics, Faculty of Medicine and Biomedical Sciences, University of Yaoundé 1, Yaoundé, Cameroon; 2 Department of Biochemistry, Faculty of Science, University of Dschang, Dschang, Cameroon; 3 Gynecology and Obstetrics Unit, Central Hospital of Yaoundé, Yaoundé, Cameroon; 4 Malaria Unit, Centre Pasteur du Cameroun, Yaoundé, Cameroon; 5 Ministry of Public Health, National Malaria Control Programme, Yaoundé, Cameroon; 6 Gyneco-Obstetric and Paediatric Hospital of Yaoundé, Yaoundé, Cameroon

**Keywords:** Malaria, Pregnancy, CareStart pf/PAN RDT, Sensitivity, Yaoundé, *Plasmodium falciparum*, Accuracy

## Abstract

**Background:**

The need to start treatment early for pregnant women who present with clinical features of malaria usually conflicts with the need to confirm diagnosis by microscopy (MP) before treatment, due to delays in obtaining results. Parasite sequestration in the placenta is also a problem. Rapid diagnostic tests (RDT), which detect soluble antigens, are a valuable alternative. The objective of this study was to evaluate pretreatment parasite prevalence by microscopy and by RDT and to assess the accuracy of RDT with MP as reference.

**Methods:**

A prospective cross-sectional study was carried out at the obstetrical unit of the Central Hospital in Yaoundé, during the period January-August 2015. Consenting patients with symptoms of suspected malaria in pregnancy were recruited and a blood sample taken for MP and RDT before treatment was started. The estimates of diagnostic performance (with 95% confidence interval) were calculated in OpenEpi online software using the Wilson’s score. The agreement, as reflected by the Cohen’s kappa, was calculated and interpreted using known intervals.

**Results:**

The results showed that, out of the 104 patients recruited, 69.2% (95%CI: 59.1–77.5) were MP positive while 77.94% (95%CI: 63.1–80.9) were RDT positive. The sensitivity of the malaria RDT was 91.67% (95%CI: 83.69–96.77) while the specificity was 53.13% (95%CI: 31.39–65.57). The diagnostic accuracy of the RDT with MP as reference was 79.81% (95%CI: 70.0–86.1). All cases were due to *Plasmodium falciparum*. A Cohen’s kappa of 0.45 (95%CI: 0.26–0.64) was obtained, consistent with a moderate agreement between the tests.

**Conclusions:**

The diagnostic accuracy of the CareStart™ malaria Pf/PAN compared to microscopy was high, but not as desirable, with a false negative RDT at very high parasitaemia. In tertiary facilities, RDTs appear to provide a better diagnostic solution compared to microscopy. However, future studies with larger sample sizes should make this observation more generalizable; as missing a case could have serious consequences on pregnancy outcome.

**Supplementary Information:**

The online version contains supplementary material available at 10.1186/s12936-022-04109-6.

## Background

About 90% of the world's malaria deaths occur in Africa [1], where most countries are hyperendemic for the disease and where 90% to 100% of cases are due to *Plasmodium falciparum* [2–4]. Pregnant women are particularly susceptible to malaria because of immunological changes and increased exposure to mosquito bites [5–8]. An estimated 10,000 women and 200,000 infants die because of malaria infection during pregnancy; severe maternal anaemia, prematurity, and low birth weight contribute to more than half of these deaths [9, 10]. Prompt and effective treatment of malaria is a crucial arm in the control of the disease, alongside a battery of preventive measures. However, it is greatly dependent on proper and timely diagnosis. According to the latest World Health Organization (WHO) guidelines, the treatment of malaria should be backed by parasitological confirmation, unless this is not feasible [11]. In endemic country context, however, laboratory analyses sometimes take longer than is desired to begin treatment, when a pregnant woman is suspected to have malaria. This is especially true when there are clinical features of severe malaria and it creates a conflict between the need for diagnosis and urgency of treatment.

The rapid diagnostic test (RDT) represents a potentially useful alternative to microscopy, but it is still necessary to assess its diagnostic value in the context of pregnancy, given a change in parasite kinetics in relation to parasite sequestration in the placenta. It is also necessary to continue evaluating these tests and to study how to cope with their expected limits, with respect to precision of parasite load, which is important in the management of the disease.

Studies on malaria parasites in pregnant asymptomatic women have reported varying rates of parasitaemia, ranging from 1.2 to 4% with 40% of asymptomatic pregnant women positive by rapid tests in one study [12–17]. However, the current study assesses parasitaemia in symptomatic pregnant women diagnosed with clinical malaria. In addition to correlating the two diagnostic modalities, microscopy and RDT, it gives a clue to the clinical diagnostic accuracy of malaria at the central hospital of Yaoundé, a typical referral facility in a malaria hyper-endemic region.

The clinical presentation of malaria depends on the causative parasite species. In addition to the cyclical rupture of red blood cells, common to all *Plasmodium* species, *P. falciparum* causes cytoadherence [18] of infected erythrocytes and rosetting, defined by adherence of infected to uninfected red blood cells [19]. This leads to plugging of small blood vessels of the brain, kidneys and other affected vital organs. The hallmark of falciparum malaria in pregnancy is parasite sequestration in the placenta, through adherence of infected red blood cells to chondroitin sulphate A [20]. These parasites evade host defense mechanisms (splenic processing and filtration) and the phenomenon perturbs normal placental function. Placental parasitization may lead to fetal growth restriction, low birth weight, maternal and fetal anaemia, while the systemic infection (with fever) may cause maternal and fetal mortality, miscarriage, and premature birth [21–23].

Cameroon is hyper-endemic for malaria with 71% of the population living in high transmission zones. About 80–100% of cases are due to *P. falciparum* and transmission is perennial in Yaoundé, with no significant seasonal variations [24]. A report of old data (1995–1998) in 2005 revealed that in Yaoundé, 27.5% of pregnant women at delivery were blood-smear positive for *P. falciparum*. Sub-microscopic infection detected by PCR was present in 54.9%. The prevalence of *Plasmodium malariae* and *Plasmodium ovale* was 7.6% and 2.5%, respectively [2]. Earlier studies found an asymptomatic smear-positive malaria prevalence of 26% and 40% in Yaoundé and Garoua, respectively [17, 24].

Parasitological diagnosis helps to improve patient care in parasite-positive patients, identify parasite-negative patients in whom other diagnoses must be sought, prevent unnecessary use of anti-malarial drugs, improve malaria case detection and reporting. Microscopy permits optimum speciation and quantification of parasites. It is also useful in the assessment of response to antimalarial treatment. Polymerase chain reaction (PCR) tests, developed lately and based on amplification of specific parasite genes, are more sensitive than microscopy and RDTs [25, 26]. Unfortunately, they are not yet available for routine clinical use. The goal of this study was to determine the accuracy of RDT in the diagnosis of malaria in pregnancy. The secondary objectives were to determine parasite prevalence among all patients clinically diagnosed of malaria in pregnancy and among those with a negative RDT.

## Methods

### Study design and setting

The study was a prospective cross-sectional assessment of the diagnostic indicators of a rapid malaria antigen-capture test against the reference, light microscopy. Sampling was consecutive and included all potential pregnant women who presented at the maternity of the Yaoundé Central Hospital between January and August 2015. The Yaoundé Central Hospital is one of the largest hospitals in the city of Yaoundé located at latitude 3°8714.

and longitude 11°5104. Founded as a day hospital, it has undergone several structural changes to become a 24/7 facility which also follows up more than 12,000 HIV/AIDS patients.

### Inclusion criteria

The following criteria were used to select participants of the study.

1. All pregnant women suspected of malaria in pregnancy at the central hospital in Yaoundé.
2. History of fever in the preceding 24 h or axillary temperature of ≥ 37.5 °C at presentation in the maternity.
3. acceptance to take part in the study. Those who accepted to take part were also required to provided informed consent.

### Exclusion criteria

Pregnant women at the maternity were excluded from participating in the study if they fulfilled at least one of the following criteria:

1. Pregnant women with history of anti-malarial treatment within the past 28 days.
2. Patients who were diagnosed of another febrile illness not related to malaria or a history of a chronic disease, such as lower respiratory tract infections, known chronic diseases, renal or hepatic diseases, HIV/AIDS.
3. Pregnant women who did not consent to the study procedures or potential publication of study results.

### Blood collection and procedure for the index test

A blood sample was taken used for malaria parasite microscopy (MP) smearing and RDT using a vacutainer needle. The RDT was done immediately at the patient’s bedside with freshly collected blood. The test kit used was the CareStart™ malaria Pf/PAN (HRP-2/pLDH) Ag Combo RDT (ACCESSBIO, Somerset, NJ, USA) obtained from the national medical commodities distributor (CENAME). These tests can detect parasite antigens, either specific to *P. falciparum* (Histidine Rich Protein 2, HRP2) or expressed by all species of *Plasmodium* (Lactate dehydrogenase, LDH). The tests were performed and interpreted in accordance with the instructions of the manufacturer. The test was conducted using anti-coagulated venous blood and the results were read within 20 min. The test was recorded as positive if both the test band corresponding to HRP2 and/or LDH, and control (C) band appeared; if only the C line was seen, it was recorded as negative according to the manufacturer ‘s instruction.

### Procedure for light microscopy, the gold standard test

The gold standard test used for accuracy analysis was the light microscopy done in the same laboratory and by the same team. For microscopy, thin and thick smear slides were prepared and stained with Giemsa (diluted 1:10 dilution with phosphate buffer) for 20–25 min. The slides were examined under oil immersion field of binocular light microscope with objective X100 (Olympus CX23). A minimum of 200 high power fields in the thick smear were read before declaring a slide negative. Quantification of parasites was done according to WHO guidelines. Those who performed the microscopy were blinded to the results of the RDT test.

### Quality control of malaria testing by microscopy

A Giemsa working solution was routinely prepared every 4–6 h to avoid deterioration of staining properties. Routine microscopy procedures followed WHO recommendations. Technicians who performed microscopy or independently reviewed study slides were WHO level 1 competent, based on WHO competency testing scheme for malaria parasite detection, identification and quantification.

The microscopy testing and crosschecking took place at the malaria laboratory of the Centre Pasteur du Cameroun, which is a regional reference centre for several biomedical investigations in Cameroon. The laboratory regularly participates in an external quality assurance programme. In addition, randomly selected 10% of patient slides were forwarded to a blinded external microscopist for assessment (the Biotechnology Centre, University of Yaoundé 1).

### Data collection

A case report form was filled for each patient through which information on pregnancy history, symptoms and signs and malaria test results were recorded. The presence of signs of severity of malaria, as defined by the WHO [27], in addition to hyperpyrexia (temperature ≥ 40 °C) and of signs and symptoms suggestive of other infections, like cough, rhinorrhea, pollakiuria and diarrhoea was noted.

### Data analysis

#### Sample size

- Sampling of pregnant women in the study was consecutive. The calculated sample size was 139 participants. Sample size was calculated using the formula: n = (Z2 × P × (1—P))/e2
- Where:
  - Z = value from standard normal distribution corresponding to desired confidence level (Z = 1.96 for 95% CI)
  - P is expected true accuracy of the index test ( at least 90%).
  - e is desired precision set at 0.05.

The sample size was estimated using Epitools (available at: www.epitools.ausvet.com.au).

All data were entered in an MS Excel 2010 spreadsheet and checked for transcription errors by two data entry operators. Data cleaning was performed, variables coded prior to statistical analysis. Sociodemographic data were presented as summaries of proportions of different categories, while continuous data such as parasite count or weight of participant were presented as means and standard deviation. The estimates of indicators of diagnostic performance were calculated in openepi online software (OpenEpi, Version 3, open source calculator) using the Wilson’s score as the method of estimation. For each proportion, the 95% confidence interval was estimated. The agreement, as reflected by the Cohen’s kappa estimator, was interpreted based on standard categories for intervals of the kappa values. Values ≤ 0 as indicating no agreement and 0.01–0.20 as none to slight, 0.21–0.40 as fair, 0.41– 0.60 as moderate, 0.61–0.80 as substantial, and 0.81–1.00 as almost perfect agreement.

### Ethical considerations

Ethical clearance was obtained from the Ethics Committee of the Faculty of Medicine and Biomedical Sciences of the University of Yaoundé 1. All patients who participated in the study provided written informed consent. The study goals, objectives, methods, and procedures were explained to the patients in the English or French language depending on the preference of the patient. The risks, benefits, as well as measures to maintain confidentiality and privacy of the data on study participants were explained to all eligible patients. Each enrolled participant provided informed consent prior to enrollment. The consent included for potential publication of study findings using anonymized dataset.

## Results

### Patient profile data

One hundred and four of 197 were recruited into this study, all aged between 16 and 42 (mean 27.19 ± 5.11). The reason for non-participation were:

1. Fulfilling at least one exclusion criteria: 77
2. Refused consent: 16.

Parity ranged from 0–5 (mean 1.26 ± 1.31). Gestational age range was 7–41 weeks (mean 29.02 ± 8.74). One patient had unknown gestational age, 5 (4.8%) were in the first trimester (< 14 weeks), 40 (38.5%) were in the second trimester (14–27 weeks) and 58 (55.8%) were in the third trimester of pregnancy (28 completed weeks or more). Their weight range was 47–120 kg (mean 75.61 ± 14.29).

Regular use of IPT (intermittent preventive treatment): patients who had received at least 2 doses of IPT or were less than 2 months from their first dose. Irregular use of IPT: patients who were more than 2 months from first dose of IPT. Non-use of IPT: patients who had not taken any dose of IPT after 18 weeks of pregnancy. Regular use of LLIN (long-lasting insecticidal nets): patients who reported sleeping under a treated bed net every night.

Most of the study participants had at least a secondary education (90.4%) and more than two-thirds were married or co-habiting. Concerning insecticide-treated bed net use, 74 patients (72.1%) reported regular bed net use, while 29 (27.9%) reported irregular or non-use. A greater proportion, 68/88 (77.3%) of the 90 pregnant women with gestational age 18 weeks and above, eligible for evaluation of IPT reported to have taken it regularly, while this information was not available for 2 patients. All of those who reported to have taken IPT used sulfadoxine-pyrimethamine (Table 1).

**Table 1:** Socio-demographic and malaria prevention characteristics among study participants

Parameter	Category	Number	Proportion (%)
Educational level	Primary and below	10	9.6
	Secondary and above	94	90.4
Civil status	Married or cohabiting	68	65.4
	Not married	36	34.6
LLITN use	Regular use	74	72.1
	Irregular or non-use	29	27.9
IPT use (eligible women)	Regular use	68	77.3
	Irregular use	9	10.2
	Non-use	11	12.5
IPT drug used	Sulfadoxine/pyrimethamine	78	100.0
	Other drug	0	0.0

### Clinical and parasitological profile of patients

The following table (Table 2) presents the clinical features of patients as well as the parasitological profile of diagnosis based on RDT and microscopy.

**Table 2:** Clinical presentation and parasitological profile of the study participants

Criterion	Number	Proportion in % (95% confidence interval)
Confirmed fever	83	79.8 (71.3–87.3)
Unconfirmed (history of) fever	21	20.2 (12.7–28.6)
Signs of severe malaria	64	61.5 (49.5–68.9)
Signs of other infection	14	13.5 (07.4–21.2)
Microscopy positivity	72	69.2 (59.1–77.5)
RDT positivity	81	77.9 (63.1–80.9)

Based on the clinical presentation, all patients had a history of fever but it was confirmed in 78.9% of cases (95%CI: 71.3–87.3). The rest of the women had a history of fever within the days preceding the hospital visit. Close to two-thirds of the pregnant women presented with one or more features of severe malaria [61.5% (95%CI: 49.5–68.9)] while a little more than one tenth had signs suggestive of other infections [13.5% (95% CI: 07.4–21.2)]. There were eight less positive cases by microscopy [69.2% (95%CI: 59.1–77.5)] than by the malaria RDT [77.9% (95%CI: 63.1–80.9)]. The geometric mean parasite load was 25,282 /µL among MP positive cases. The only *Plasmodium* species identified, based on microscopy and RDT was *P. falciparum*. It was found that 17 of the 32 MP negative patients (53.1%) were also RDT negative and that five of the 19 RDT negative cases were microscopy positive with parasite densities at the extremes of the parasite count and geometric mean 2,473/µL (Additional file 1: Table S1).

### Accuracy parameters of the malaria rapid diagnostic test.

The indicators of diagnostic performance of the malaria RDT were calculated and summarized in Table 3. Light microscopy was used as the reference test (see Additional file 1: Table S3). Table 3 shows that RDT sensitivity was 91.67% (95%CI: 83.69- 96.77) while the specificity was 53.13% (95%CI: 31.39–65.57). The predictive value of the positive test was also high, but as expected the predictive value of the negative test was slightly lower [73.91% (95%CI: 51.21–88.19)]. The overall accuracy of the RDT was estimated at 79.81% (95%CI: 70.00–86.09). The likelihood ratio of the positive test was less than 2 and the test agreement, as reflected in the estimation of the Cohen’s kappa, was moderate [0.45 (95%CI: 0.26–0.64)].

**Table 3:** Diagnostic value of RDT with MP as reference

Parameter	Estimate	Lower—Upper 95% Cis
Sensitivity	91.67%	82.99–96.12
Specificity	53.13%	36.45–69.13
Positive Predictive Value	81.48%	71.67–88.44
Negative Predictive Value	73.91%	53.53–87.45
Diagnostic Accuracy	79.81%	71.10–86.40
Likelihood ratio of a Positive Test	1.95	1.71–2.23
Likelihood ratio of a Negative Test	0.15	0.10–0.24
Diagnostic Odds	12.47	4.20–36.95
Cohen's kappa (Unweighted)	0.48	0.29–0.67

### Parasite load among those with signs suggestive of other infections

Among the 14 pregnant women with signs suggestive of other infections, eight were positive for malaria by light microscopy and their mean parasite load was 1,896 trophozoites of *P. falciparum (*TPf)/uL, with a range of 457–10,000/uL. The same number, eight, was positive for RDT. Discrepant results were found in four cases. Two (14.3% of the 14 women) had negative RDThe sample T and positive MP (mean parasite load 457/uL) and 2 with negative MP had a positive RDT. Overall, seventeen women were negative for both MP and RDT, and only four of them (23.5%) had no symptoms or signs suggestive of other infection.

## Discussion

The goal of this study was to determine how well a malaria RDT test performs in the diagnosis of malaria in febrile pregnant women in a tertiary health care facility in Cameroon. The study sample, composed of 104 pregnant women consulting at the maternity of the Central Hospital in Yaoundé, showed diversity in terms of maternal age, parity and gestational age, suggesting good representation. The high level of education with 90% of patients having attained secondary level of education or higher also reflects the general population in the urban setting of Yaoundé.

The importance of IPT and LLITN in the prevention of malaria in pregnancy has been reported [28]. The declared 72.1% regular use of LLITN and 77.3% regular IPT use – defined by at least 2 doses received or patient less than 2 months from first dose – among the patients suggests awareness about malaria is significant but the limits of these methods of prevention are real. Parasitological failure rates of IPT with SP as high as 26% have been reported [29, 30]. Other studies have associated high failure rates of IPT and LLITN use with young age (15–19 years), low parity, single status and low level of education [31, 32]. However, up to 28% of pregnant women in this study did not use LLITN regularly and close to 23% were not regular with IPT. This may reflect suboptimal sensitization during antenatal consultation (ANC) but may just be the reflection of a selection bias, given that those who do not use these preventive measures may be more likely to have malaria and come to hospital where the recruitment took place. Therefore, low uptake of SP or LLITN among the study patients might not reflect the true situation in the community.

All our patients reported fever and it was confirmed in 79.8% of them, illustrating the value of fever as a key clinical feature of malaria in pregnancy in malaria endemic settings. More than half (61.5%) of our patients showed clinical signs of severity including hyperpyrexia (temperature of 40 °C and above). Although this does not figure on the WHO criteria for severity of malaria of 2010, it may be considered crucial in the context of pregnancy, where high temperatures could lead to fetal death.

The accuracy of RDT with respect to MP (79.81%) is only slightly less than the 82.9% reported [33]. The sensitivity (91.67%) and, especially, the specificity (53.13%) observed in this study, however, are less satisfactory with respect to the 96.8% and 73.5%, respectively reported in a study carried out in Uganda for the same HRP2 RDT test, among 433 pregnant women with fever [34]. The proportion of patients with positive parasitology was slightly higher with RDT than with MP. This trend has already been reported among asymptomatic pregnant women in Burkina Faso (47% for RDT versus 30% for MP) [12]. However, when the two tests were assessed, with PCR as reference, MP had a sensitivity of 94.4% and RDT 83.3%. The specificities were 100% for MP and 92% for RDT [12]. Therefore, the results obtained may reflect greater sensitivity or, more probably, a weaker specificity and Positive Predictive Value (PPV) for the RDT.

The geometric mean parasite load (2.73/uL) among the patients with negative RDT is high but much less than the value among those with a positive RDT (27,227/uL). Patients with signs compatible with other infections had a sharp decrease in positive test results for RDT and MP (8/14 or 57% for both) and the average parasite load among those with positive MP dropped to 3,971/uL. These suggest a false clinical diagnosis for malaria in some of these cases. The low MP positive rate (1.9%) and low mean parasite load (457/uL) among patients with negative RDT and presence of signs suggestive of other infection favors this combination of arguments to rule out malaria in most of these cases. Submicroscopic malaria infections appear to be common in pregnancy, especially among the primigravidae as seen in recent studies [4, 26, 35]. Indeed, in the studies mentioned, the authors found more submicroscopic infections during pregnancy than microscopic infections. Although no attempt to identify submicroscopic infections was made in our study, the analysis of the performance of both tests, with up to 32 patients negative by MP, and 14 patients negative for both tests, highlights the possibility of the presence of submicroscopic parasitaemia among study participants. There is, therefore, a need to determine, by some reference test such as PCR or the Loop-mediated isothermal amplification (LAMP) methods, the proportion of pregnant patients with negative RDT and MP, who could still have malaria [36]. This would highlight the importance of adherence to preventive measures during pregnancy, to avoid adverse pregnancy outcomes [37–40].

The geometric mean parasite load among MP positive subjects was high (125,587 TPf/uL). However, this is about half the parasite load considered hyperparasitaemia for a malaria hyperendemic area [11]. Only *P. falciparum* was observed by microscopy and none of the PF + Pan RDT was positive for 'Pan ‘ and negative for 'Pf ‘, implying that none of these tests was positive for monoinfection with species other than *P. falciparum*. This is not consistent with earlier findings in the same setting and earlier reports of non-falciparum placental malaria, especially due to *P. malariae* [24, 41], and it could reflect a change in parasitology in response to the interventions that have been implemented of recent (IPT with SP, and LLITN).

One of the five patients with negative RDT and positive MP, had a parasite load that was too high to be counted. This ‘prozone effect’ has been reported in earlier studies [42]. It may also be a reflection of parasites with deletions in the *hrp*2/3 genes, as has been shown in previous studies [43] and needs to be investigated further in our setting. These false negative results, coupled with the observation that only 2 of 14 women (14.3%) who were negative for MP and RDT, had no sign suggestive of other infection suggests a high combined sensitivity for the two tests. That is, 86.7% of patients with fever who were negative for both tests had a sign suggestive of other infection. So, doing both tests for each patient may help to avoid missing a malaria case.

These results should, however, be interpreted with some caution. Firstly, the small sample size of this study. It would have been desirable to have larger sample size to improve on the study power, although in prospective designs with large sample size requirement, cost may be a limiting factor. However, this study took place in the maternity of a tertiary facility where not as many malaria suspected pregnant patients are seen compared to district or primary health centers. Secondly, the number of symptomatic volunteers suspected for malaria among pregnant women is a smaller proportion of all symptomatic patients suspected for malaria in most health facilities in Cameroon. Thirdly, the specificity of the RDT test could be different (better) if the sample size was larger and design allowed for more sampling of pregnant women in the hospital outpatient and at antenatal care. Therefore, in the context of this study, the accuracy would seem to be biased towards positive cases. In addition, a significant number of pregnant women was excluded, some of whom should have been part of the study were they tested. They included symptomatic pregnant women who self-medicated before visiting at the hospital; or who were diagnosed for other febrile conditions than malaria, further limited the number of negative cases. Therefore, this study sample reflected a typical scenario under the local malaria management conditions. Future studies will consider these factors carefully when estimating an appropriate sample size for diagnostic evaluations in this special patient population.

## Conclusion

This study has shown slightly higher positivity for malaria RDT than for microscopy in pregnant women presenting with fever in a tertiary care setting in Cameroon. This may not, however, necessarily mean greater sensitivity, as patients with very high parasite densities could potentially present negative RDT results. Owing to the advantages of promptitude of results and little need for laboratory, RDT is recommended for facility diagnosis of malaria among pregnant women in the maternity. Studies with larger sample sizes are needed to make the findings more generalizable; as missing a case in pregnancy could have serious consequences on maternal or child health.

## Supplementary Information

**Additional file 1: Table S1.** Parasite load with respect to TDR and MP results.** Table S2.** MP and TDR among patients with signs compatible with other infections.

## Data Availability

All data generated or analysed during this study are included in this published article [and its additional information files].
