# Supplementary material for: Diagnosis of malaria in pregnancy: accuracy of CareStart™ malaria Pf/PAN against light microscopy among symptomatic pregnant women at the Central Hospital in Yaoundé, Cameroon
Source: Malar J. 2022 Mar 9;21:78. doi: 10.1186/s12936-022-04109-6 (PMC8905860; doi:10.1186/s12936-022-04109-6)
Supplement: Supplementary file 1 — Additional file 1: Table S1. Parasite load with respect to TDR and MP results. Table S2. MP and TDR among patients with signs compatible with other infections. [file 12936_2022_4109_MOESM1_ESM.docx]

**Additional tables**

Additional tables

**Table S1**: Parasite load with respect to TDR and MP results

| Scenario | Number | Frequency(%) | Geometric Mean Parasite load/µL (range) |
| --- | --- | --- | --- |
| MP +, TDR + | 66 | 63.4 | 27,227.11  (163 – 300,000) |
| MP +, TDR - | 6 | 5.8 | 2,473 (457 – 300,000) |

**Table S2**: MP and TDR among patients with signs compatible with other infections.

| Test | Number | Positive | Frequency (%) |
| --- | --- | --- | --- |
| MP | 14 | 8 (Geometric mean = 1,896 /uL) | 57 |
| RDT | 14 | 8 | 57 |
